# Clinical evaluation of accelerated cardiac cine imaging using iterative k-t-sparse SENSE

**DOI:** 10.1186/1532-429X-16-S1-W13

**Published:** 2014-01-16

**Authors:** Bradley D Allen, Maria Carr, Michael O Zenge, Michaela Schmidt, Mariappan S Nadar, Bruce S Spottiswoode, Jeremy D Collins, James C Carr

**Affiliations:** 1 Radiology, Northwestern University, Chicago, Illinois, USA; 2 Siemens AG Healthcare Sector, Erlangen, Germany; 3 Siemens Corporate Technology, Princeton, New Jersey, USA; 4 Siemens Healthcare USA, Inc., Chicago, Illinois, USA

## Background

We sought to quantitatively and qualitatively evaluate a clinical implementation of accelerated MR acquisitions using iterative segmented k-t-sparse Cartesian SENSE balanced steady-state free-precession (SSFP) cinegraphic imaging.

## Methods

**Figure 1:**
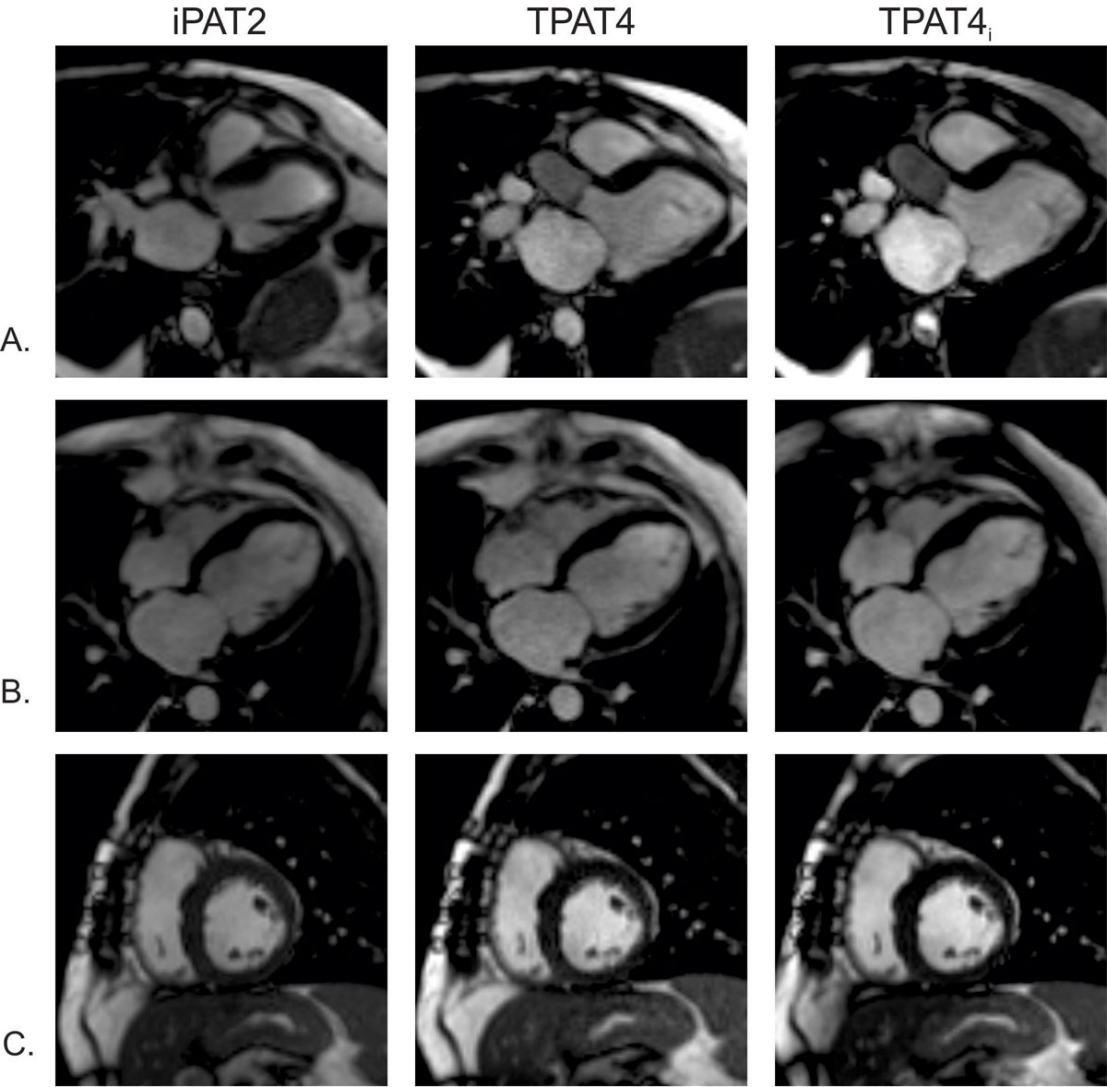
**Example A) three-chamber, B) four-chamber, and C) mid-chamber short axis images in a patient undergoing CMR for aortic valve assessment using each acquisition technique**.

IRB approval was obtained. Twenty patients (age: 54.8 ± 14 years, M:F = 15:5) undergoing non-emergent CMR assessment for myocardial pathology were consecutively recruited. Nine healthy volunteers (age: 44.3 ± 14 years, M:F = 6:3) were also imaged. CMR was performed at 1.5T (MAGNETOM Aera, Siemens Healthcare, Erlangen, Germany). The examination included acquisition of standard segmented SSFP (iPAT2) (GRAPPA accel factor 2, TR 40 msec, 2.1 × 2.1 × 10 mm^3^) cine and two accelerated segmented SSFP acquisitions (TPAT accel factor 4, TR 37.7 msec, 2.1 × 2.1 × 6 mm^3^), one with an investigational prototype inline iterative k-t-sparse SENSE reconstruction with L1 regularization along one spatial and temporal dimension (TPAT4_i_) (1)and the other with conventional SENSE reconstruction (TPAT4). Each technique was used to acquire a three- (3Ch), four-chamber (4Ch), and short axis (SA) series in identical slice positions (Figure 1), with SA coverage of the entire left ventricle (LV) with 10 mm interslice gaps. Individual slice scan times were recorded. Quantitative LV functional analysis was performed. A reviewer blinded to acquisition type scored images for overall image quality, noise, and artifacts using a 5-point Likert scale. Continuous variables were compared between groups using a paired t-test, and ordinal variables were compared using a Wilcoxon signed-rank test.

## Results

**Figure 2:**
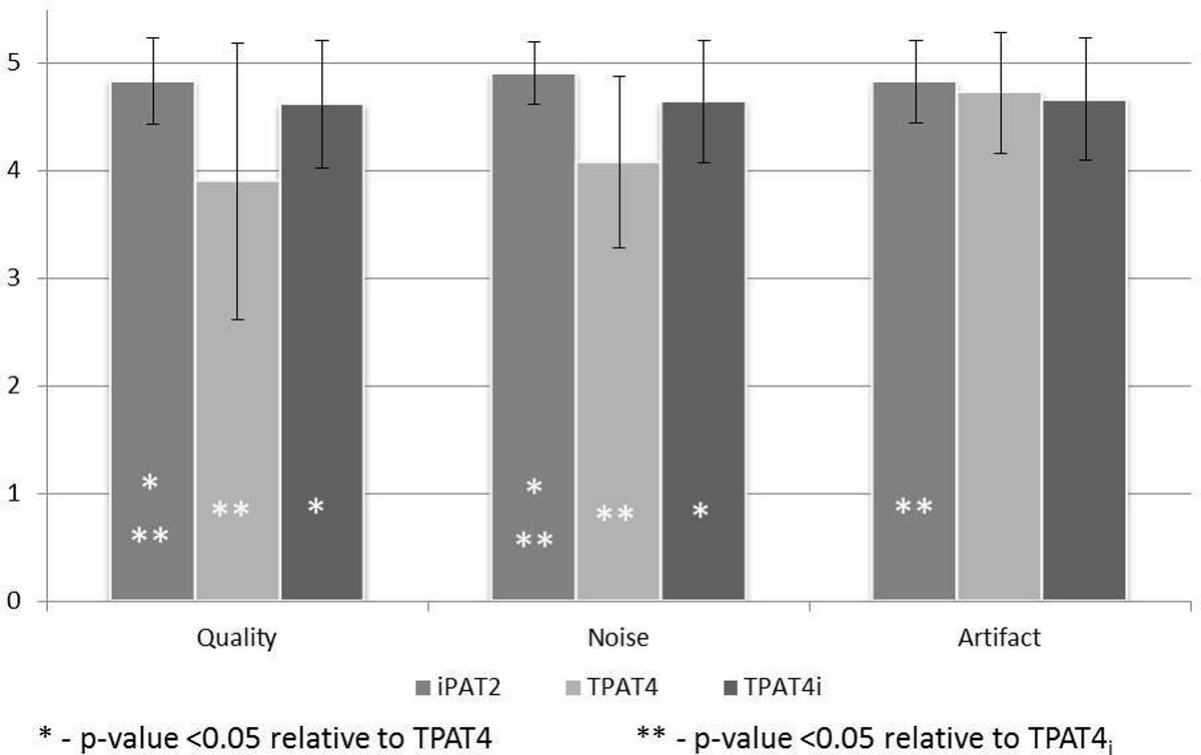
**Qualitative scoring in the combined cohort of patients and volunteers**.

In a combined analysis of patients and volunteers, there was no significant difference between LV ejection fraction between iPAT2 and TPAT4_i _(p = 0.48) or TPAT4 (p = 0.34). The iPAT2 technique found marginally lower end-diastolic volume (159 ± 54 ml vs. 169 ± 54 ml, p = 0.01), end-systolic volume (75 ± 33 ml vs. 80 ± 31 ml, p = 0.04), stroke volume (80 ± 35 ml vs. 89 ± 31 ml, p = 0.03) compared to TPAT4_i_. Single-slice scan times in both TPAT4_i_(3.29 ± 0.6 sec) and TPAT4 (3.0 ± 0.6 sec) acquisitions were significantly shorter relative to iPAT2 (8.4 ± 1.7 sec, p < 0.001 for both). Qualitative review showed significantly higher image quality and lower noise using both iPAT2 and TPAT4_i _acquisitions relative to TPAT4 (quality: p < 0.001, noise: p < 0.001). The iPAT2 acquisition was scored higher than TPAT4_i _in quality (4.8 ± 0.4 vs. 4.6 ± 0.6, p = 0.005), noise (4.9 ± 0.3 vs. 4.6 ± 0.6, p < 0.001), and artifact (4.8 ± 0.4 vs. 4.6 ± 0.6, p = 0.04), although the magnitude of difference is relatively small. (Figure 2)

## Conclusions

Acceleration using iterative k-t-sparse SENSE techniques can be successfully applied in CMR to reduce scan times by > 50% while maintaining diagnostic image quality and quantitative accuracy in LV systolic function assessment.

## References

[B1] Liu J ISMRM 20th Annual Meeting 2012 Melbourne, Australia 4249

